# Ovulatory Follicular Fluid Facilitates the Full Transformation Process for the Development of High-Grade Serous Carcinoma

**DOI:** 10.3390/cancers13030468

**Published:** 2021-01-26

**Authors:** Che-Fang Hsu, Pao-Chu Chen, Vaishnavi Seenan, Dah-Ching Ding, Tang-Yuan Chu

**Affiliations:** 1 Center for Prevention and Therapy of Gynecological Cancers, Department of Medical Research, Hualien Tzu Chi Hospital, Buddhist Tzu Chi Medical Foundation, Hualien 970, Taiwan; CFHsu@tzuchi.com.tw (C.-F.H.); 104727118@gms.tcu.edu.tw (V.S.); 2 Department of Obstetrics & Gynecology, Hualien Tzu Chi Hospital, Buddhist Tzu Chi Medical Foundation, Hualien 970, Taiwan; coral@tzuchi.com.tw (P.-C.C.); dah1003@tzuchi.com.tw (D.-C.D.); 3 Institute of Medical Sciences, Tzu Chi University, Hualien 970, Taiwan; 4 Department of Life Sciences, Tzu Chi University, Hualien 970, Taiwan

**Keywords:** follicular fluid, high-grade serous carcinoma, Fallopian tube, transformation, peritoneal metastasis

## Abstract

**Simple Summary:**

Ovulation is regarded as the culprit of ovarian high-grade serous carcinoma (HGSC). Previously, we discovered IGF2 in the ovulatory follicular fluid (FF), which bathe fallopian tube epithelium (FTE) during ovulation, promotes malignant transformation through the IGF-1R/AKT pathway. Transformed FTE cells then exfoliate and metastasize to the ovary and peritoneum to grow overt HGSC. In this study, we utilized immortalized FTE cells and HGSC cells carrying accumulating severity of driver mutations to explore FF’s role in the development of HGSC. We found FF promotes (by order of magnitude) migration, anchorage-independent growth (AIG), invasion, peritoneum attachment, anoikis resistance, and proliferation of the full panel of tested cells. The AIG activity largely depends on IGF-1R/AKT signaling, and both AKT- and non-AKT-mediated signals are responsible for other phenotypes. The results demonstrated an extensive transformation activity of FF in the full journey of HGSC development from FTE.

**Abstract:**

*Background*: High-grade serous carcinoma (HGSC) is mainly derived from the stepwise accumulation of driver mutations in the fallopian tube epithelium (FTE), and it subsequently metastasizes to the ovary and peritoneum that develops into a clinically evident ovarian carcinoma. The developmental process involves cell proliferation/clonal expansion, cell migration, anoikis resistance, anchorage-independent growth (AIG), peritoneum attachment, and cell invasion. Previously, we discovered FTE could be transformed by follicular fluid (FF) released from ovulation, the most crucial risk factor of ovarian cancer, and IGF axis proteins in FF confers stemness activation and clonal expansion via IGF-1R/AKT pathway. However, whether other phenotypes in advanced cancer development are involved is unknown. *Methods*: A panel of FTE and ovarian HGSC cell lines with different severity of transformation were treated with FF with or without IGF-1R and AKT inhibitors and analyzed for the transformation phenotypes in vitro, ex vivo, and in vivo. *Results*: FF largely promotes (by order of magnitude) cell migration, AIG, cell invasion, peritoneum attachment, anoikis resistance, and cell proliferation. Most of these activities worked in the full panel of cell lines. The AIG activity largely depends on IGF-1R/AKT phosphorylation, and the proliferation activity depends on an AKT phosphorylation not mediated by IGF-1R. In contrast, both AKT- and non-AKT-mediated signals are responsible for the other transformation activities. *Conclusions*: Our data demonstrate an extensive transformation activity of FF in the full journey of carcinogenesis, and endorsed ovulation-inhibition for the prevention and AKT-inhibition for the treatment of ovarian HGSC.

## 1. Introduction

High-grade serous carcinoma (HGSC) is the most common and lethal subtype of epithelial ovarian cancer. A majority of patients are diagnosed at late stages with advanced intraperitoneal metastasis. After standard treatments with surgery and chemotherapy, these advanced stages of tumor show recurrence, leading to a poor patient prognosis, with a five-year survival rate of only 25–30% [1,2]. The fundamental cause of this poor outcome is the lack of knowledge about tumor etiology and the mechanism of tumor development.

Cumulative evidence has revealed that majority of HGSC originates from the epithelium of the fallopian tube, especially at the fimbria, which directly exposes to the follicular fluid (FF) during ovulation [3,4,5]. Breast cancer (BRCA1/2) gene carriers who underwent prophylactic salpingo-oophorectomy were shown to have precursor lesions with TP53 mutation, including p53 signature, serous intraepithelial lesion (STIL), and less commonly, serous tubal intraepithelial carcinoma (STIC) in the fallopian tube epithelium (FTE) [6,7]. These tubal p53 lesions were also found in patients with ovarian HGSC who underwent surgery [8] and showed a clonal relationship with concurrent HGSC lesions [9,10,11]. The natural history of the stepwise development of these tubal precursor lesions to HGSC has been estimated to take more than 30 years [12]. It includes approximately 10 years for FTE to develop a p53 signature, another 15 years to transform into STIC, and a final six to seven years to metastasize to the ovary and peritoneum to develop into a clinically observable HGSC [10,11,12].

Furthermore, intraluminal shedding of tumor cells is frequently observed in tubal STIC [13,14,15]. Indeed, exfoliated STIC cells are frequently found in the lumen of the fallopian tube [15]. This demonstrates the following model of STIC metastasis: a long-term, constant shedding of STIC cells into the peritoneal cavity, which leads to anoikis until tumor cells have evolved to survive, ability of attachment and growth in the new microenvironment on the peritoneal or ovarian surface, and invasion into the underlying stroma to establish metastasis [16]. This characteristic shedding-and-seeding mechanism of STIC metastasis differs much from the classical invasion-and-intravasation mechanism of other in situ carcinomas, and this will be a focus of our study. 

Large-scale epidemiological studies have shown that excessive numbers of ovulation (or incessant ovulation) is the most important etiological risk factor for epithelial ovarian cancer, including HGSC [17,18,19]. During ovulation, the fallopian tube fimbria juxtaposes the ovulation site and directly exposes to the fluid released from the ovarian follicle. We have previously reported multiple evidences supporting the transformation activity of FF [20,21,22]. When injected directly into the mammary fat pad of *Trp53*-null mice, FF induced early-onset lymphoma in a reactive oxygen species (ROS)-dependent and tissue-specific manner [20,22]. Addition of FF to different immortalized human fimbrial epithelial (FE) cell lines induced the transformation phenotypes, including anchorage-independent growth (AIG) and tumorigenesis in NSG mice [20,21,22]. The transformation activity is largely contributed by insulin-like growth factor 2 (IGF2) and the associated proteins, which are abundantly present in the FF. The IGF-1R/protein kinase B (AKT)-mediated signaling pathways are responsible for the transformation, AKT/mTOR is responsible for AIG and AKT/NANOG is responsible for stemness activation, clonal expansion, and also AIG [22]. Importantly, the transformation activity of FF may not be limited to IGF2 signaling and associated phenotypes. Indeed, depletion of IGF axis proteins from FF or inhibition of IGF-1R by short hairpin RNA (shRNA) or inhibitors did not completely abolish the transformation activity of FF [22]. 

For a comprehensive understanding of the role of FF in the full course of tubal carcinogenesis, the present study aimed to investigate the effect of FF on FE cells representing different severity of malignant transformation as well as fully transformed HGSC cells. To this end, mechanistic steps relating to the shedding-and-seeding mechanism of metastasis, such as cell migration, anoikis resistance, attachment growth and invasion, were analyzed. The result showed that ovulatory FF has a wide range of transformation activity in all phenotypes tested, and in all FE and HGSC cells tested. Mechanistically, IGF/IGF-1R/AKT plays a major role in early transformation, whereas other AKT-mediating growth signals not mediated by AKT are crucial for the late transformation phenotypes.

## 2. Results

### 2.1. FF Promotes Intraperitoneal Tumorigenesis of HGSC Cells and Transformation of Human Fimbrial Epithelial Cells 

We used three immortalized human fimbrial epithelial cell lines and two HGSC cell lines for this study. They represented different severities of step-wise accumulation of driver mutations during the development of HGSC from FTE. FT282-V carries a gain-of-function dominant-negative R175H mutation of the *TP53* gene. It simulates p53 signature, the earliest tubal precursor lesion with *TP53* mutation. FT282-CCNE1 adds a second hit, *CCNE1* overexpression, to disrupt the cyclin-dependent kinase inhibitor 2A (CDKN2A)/CCNE/retinoblastoma (RB) pathway. CCNE1/RB disruption occurs early in tubal secretory cell transformation and was frequently observed in STIC lesion [23,24]. In FE25 cells, p53 and RB were disrupted by E6 and E7 HPV16 oncoproteins, respectively [20]. In addition, E6 and E7 also interacted with other cellular proteins [25] and exerted diverse effects, including epigenetics [26]. OVSAHO was derived from the intra-abdominal metastasis of a patient with HGSC. In addition to TP53 and RB mutations, OVSAHO has additional neurofibromin 1 (*NF1*) mutation disrupting the NF1/KRAS) signal and fibroblast growth factor receptor 4 (FGFR4) amplification [27]. In contrast, KURAMOCHI has TP53 mutation and KRAS, MYC, and FGFR1 amplifications, but does not have RB disruption [27,28,29] (Table 1).

**Figure 1:**
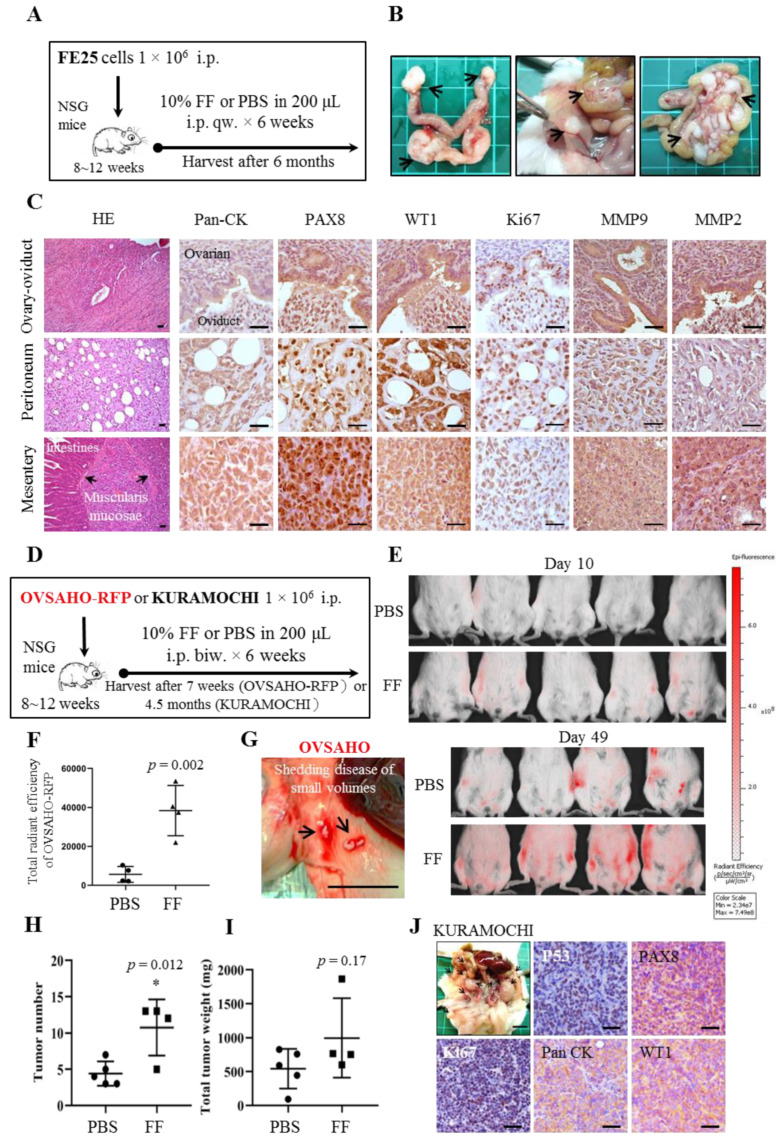
FF promotes intraperitoneal tumorigenesis of human immortalized fimbrial epithelial cells and HGSC cells. NSG mice were injected intraperitoneally with 1 × 10^6^ FE25 (**A**–**C**), OVSAHO (**D**–**G**) or KUROMACHI (**H**–**J**) cells. (**A**,**D**) Ten percent FF or PBS was co-injected with the cells and was given as boosters weekly or twice weekly for six weeks. Mice were sacrificed by 6 months, 7 weeks or 4.5 months, respectively. (**B**) Representative intraperitoneal tumor growths (arrows) on the ovary and omentum (left), the mesentery and parietal peritoneum (middle), and the forming of mesentery-omentum cake (right) were shown. (**E**) Tumor growths from RFP-labeled OVSAHO cells (OVSAHO-RFP) were detected by using the IVIS imaging system. (**C**,**J**) Histology of representative tumors was shown by hematoxylin and eosin stain and immunohistochemistry with antibody against Pan-CK, P53, PAX8, WT1, Ki67, and/or MMP2/9. Scale bars represent 50 μm. (**F**) The total radiant efficiency of OVSAHO-RFP intraperitoneal tumors was compared between the PBS- and FF-injection groups. (**G**) The OVSAHO-RFP xenograft demonstrated low-volume seedings on the peritoneum. (**H**,**I**) Numbers and total weight of the KURAMOCHI intraperitoneal tumors in the PBS- and FF-injection groups. The asterisk represents a comparison of the vehicle. * *p* < 0.05, by two-sided, unpaired Student’s *t*-test.

The transformation activity of FF was first tested in xenograft tumor models using partially transformed human fimbrial epithelial cells and HGSC cells. Aliquot from a pool of twenty-five human FFs with tumor cells was co-injected intraperitoneally into NOD/Shi-scid/IL-2Rγ (NSG) mice and boosted with FF once or twice weekly for 6 weeks (Figure 1A,D). This is to mimic the incessant ovulation scenario wherein mouse oviduct epithelium is exposed to FF every 4–5 days. No tumor growth was observed in six mice grafted with FE25 cells with PBS injection, whereas four (33%) out of twelve mice with FF injection showed tumor growth in six months. These tumors had diffuse and aggressive growth in the peritoneal cavity, mainly localized near the injection site at the mesentery/intestinal serosa (4/4) and the peritoneal wall (2/4). Interestingly, three mice had tumor growth in the ovary (Table S1). Histology of HGSC tumor revealed positivity for Pan-CK, markers of Mullerian epithelium (PAX8, WT1) and Ki67, as well as the markers of aggressiveness, matrix metallopeptidase (MMP) 9 and MMP2 [31] (Figure 1B,C). For the generation of OVSAHO xenograft models, we referred to the methods described by Elias et al. [32] and injected less number (1 × 10^6^) of cells. During the boost dose with PBS or FF, the mice showed signs of weight loss, emaciation, and died in the seventh week. Subsequently, the experiment was terminated and implants of the RFP-labeled tumor cells were observed using the IVIS system. The tumor incidence rates in both PBS and FF groups were 100% (PBS group 4/4 and FF group 4/4). Compared to the PBS control, FF-coinjected mice displayed more potent and diffuse implants (Figure 1E), with a 6.8-fold increase in total fluorescence intensity (Figure 1F). As described by Elias et al., OVSAHO tumors showed small volume seedings and implants on the surface of organs and peritoneum (Figure 1G), and all mice showed small early bowel obstructions (Figure S1A). Moreover, intravascular metastasis can be easily found in the serosa of the lesser omentum (Figure S1B,C). In the KURAMOCHI xenografts models, grossly obvious tumors were observed. Compared to control, FF injection resulted in more tumors (Figure 1H), metastasis, and invasion to the parenchyma of distant organs such as the liver, spleen, lesser omentum, and the diaphragm (Table S1). Interestingly, the increase in total tumor weight was not statistically significant (Figure 1I). Moreover, one of the FF mice (1/4) had tumor development in the ovary. Histological features and markers of HGSC were also confirmed (Figure 1J). 

Given the fact that FF enhances tumorigenesis of both immortalized FTE and HGSC cell lines, we aimed to investigate the transforming activity of FF regarding at different phenotypes.

### 2.2. FF Induces Phosphorylation of IGF-1R/AKT With Subsequent Increase of AIG

**Figure 2:**
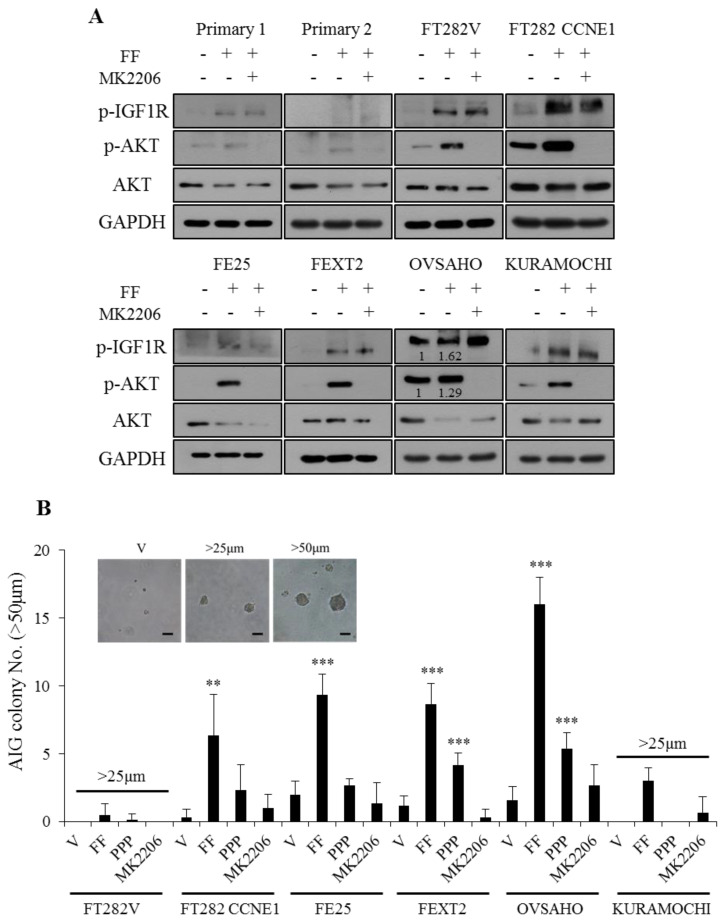
FF promotes anchorage-independent growth of FTE /HGSC cells in correlation with the phosphorylation of IGF-1R /AKT. (**A**) IGF-1R, AKT and their phosphoproteins in primary FTE cells, transforming FTE cells and HGSC cells with or without FF and/or AKT inhibitor (MK2206). Cells were pretreated for 30 min with MK2206 (10 µM) or vehicle before the addition of 10% FF or PBS. Western blot was performed after 30 min. (**B**) AIG of the six cells with the same pretreatments. Colonies over 50 µm (more number of transformed cells) or over 25 µm (less number of transformed cells) were counted after 14 days culture. Scale bar, 50 μm. Results are from three independent experiments, the asterisk represents comparison of vehicle. ** *p* < 0.01, *** *p* < 0.001 by two-sided, unpaired Student’s *t*-test. Full uncropped Western Blot images are available in Figure S2.

We first analyzed whether the previously identified transformation signaling of FF, i.e. IGF2/IGF-1R/AKT, is activated by FF. Before FF treatment, an increasing trend of baseline AKT phosphorylation was observed under serum-free culture conditions in the tested cell panel. It was mainly observed in the two HGSC and in FE282-CCNE1 cell lines. The two HGSC cell lines also showed baseline IGF-1R phosphorylation, which suggests the establishment of an IGF autocrine loop (Figure 2A). 

After FF treatment for 30 min, each of the transforming FE cells, and primary FE cells to a lesser extent, exhibited a substantial increase in IGF-1R and AKT phosphorylation as well as AIG. Importantly, IGF-1R/AKT phosphorylation induced by FF correlated well with the number of colonies in AIG (Figure 2A,B). Moreover, inhibition of IGF-1R largely diminished, and inhibition AKT inhibition almost totally abolished, the FF-induced AIG in all the tested cells. Overall, the results indicate that FF promoted AIG largely through the IGF-1R and AKT signaling pathways.

### 2.3. FF Enhanced Cell Proliferation Through Non-IGF-1R Dependent AKT Signaling

**Figure 3:**
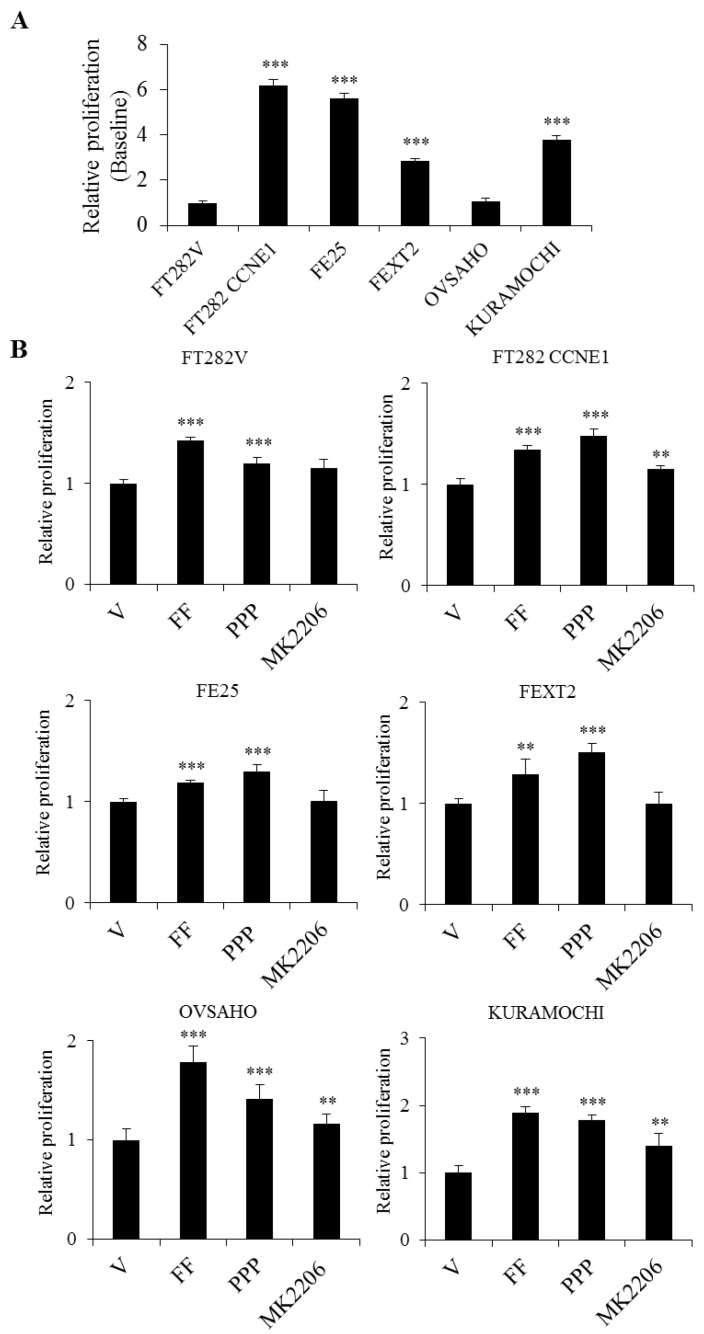
FF increases proliferation of transforming FTE /HGSC cells largely by AKT-signaling but not through IGF-1R. Under serum free conditions, cell proliferation was tested by XTT assay at baseline (no treatment) (**A**), and after treatment with vehicle, 10% FF (10%), FF + IGF-1R inhibitor (PPP) or FF + AKT inhibitor (MK2206) (**B**). Cells were harvested after 24 h. Results are from three independent experiments Error bar represents mean ± SD (*n* = 8). The asterisk represents comparison of vehicle. ** *p* < 0.01, *** *p* < 0.001 by two-sided, unpaired Student’s *t*-test.

As shown in Figure 3, FF moderately increased the proliferation of the four FTE cell lines (from 18% to 43%) and the two HGSC cell lines (from 78% to 90%). As revealed by the results of inhibitor treatment, this mitogenic activity largely depended on AKT but not on IGF-1R. The result indicated a moderate mitogenic activity of FF in the course of HGSC development, largely mediated via AKT and not via IGF-1R.

### 2.4. FF Highly Increases Matrix Attachment, and Enhances Anoikis Resistance, Partly Dependent on AKT

**Figure 4:**
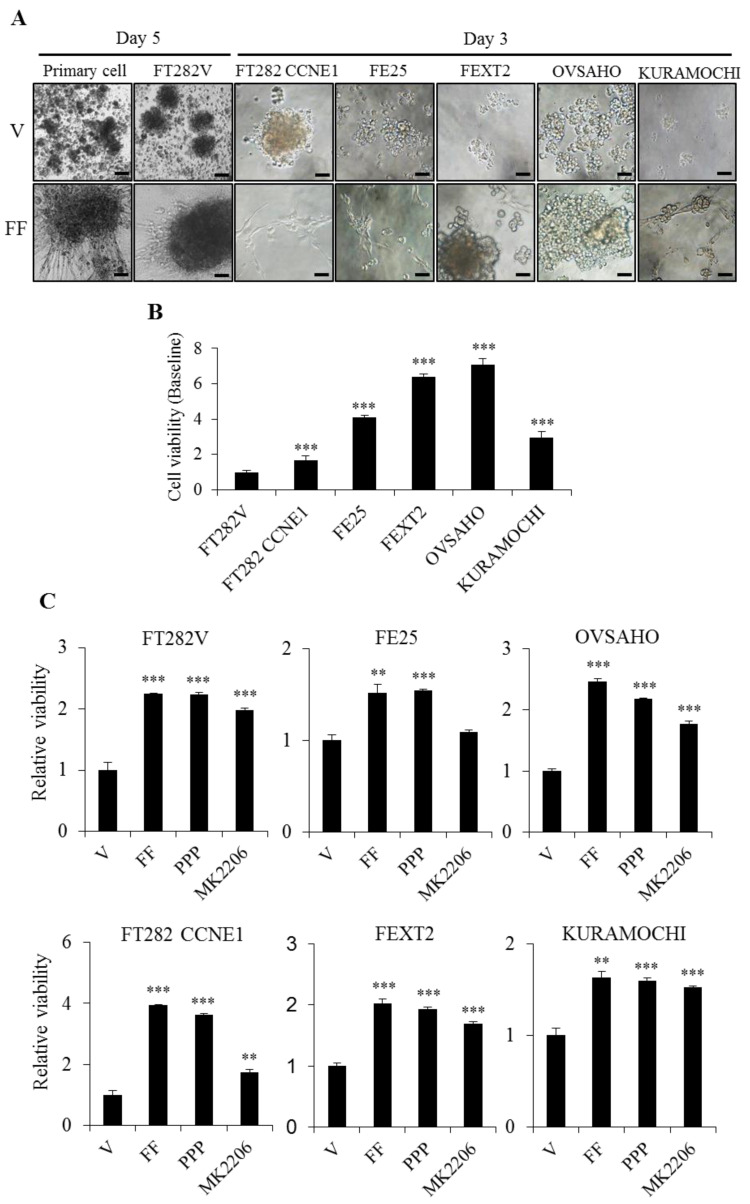
FF increases anoikis resistance in transforming FTE /HGSC cells, partly conferred by AKT. (**A**) Under serum-free conditions, cell attachment was first tested on the ultra-low attachment hydrogels with vehicle (V) or 10% FF for three or five days. Representative pictures showed remarkable cell adhesion and spreading of all cells tested after FF treatment. Scale bar, 50 μm. (**B**) In the modified anoikis assay, we used 0.4% agarose to incubate 4 × 10^3^ cells in ultra-low attachment plates for 3D suspension culture and used XTT colorimetry to detect cell viability after 24 h. Survival of FT282-V was used as the reference to compare survival of different cells. (**C**) Relative cell viability was compared in the same cell panel with or without IGF-1R/AKT inhibitor pretreatment and FF/vehicle treatment. Assays were performed in triplicates. The error bar represents mean ± SD (*n* = 8), the asterisk represents comparison of vehicle treatment. ** *p* < 0.01, *** *p* < 0.001 by two-sided, unpaired Student’s *t*-test.

In order to test the anoikis resistance activity of FF, a hydrogel coated ultra-low attachment plate was first applied in serum-free culture. Due to an extremely high attachment-enhancing activity of FF, all three transforming FTE and HGSC cells became tightly attached to the plate in 72 h after FF treatment. The non-transformed primary FE cells and FT282-V cells also showed a delayed attachment after five days (Figure 4A). To overcome this, cells were mixed with 0.4% agarose gel and placed on the top of a 0.8% agarose gel-coated ultralow-attachment plate. By this design, cells were floating in the top gel and prohibited from attachment to the bottom matrix by the high-percentage lower gel. The cell viability (anoikis resistance) correlated with the severity of transformation (Figure 4B). FF largely increased viability in all cell lines tested. Inhibition of IGF-1R had little change of the effect on viability, and AKT inhibition variably reduced the effect in different cell lines with FT282-CCNE1 and FE25 cell lines showed higher effect (Figure 4C). Thus, AKT-mediated signaling is partly responsible for the anoikis resistance activity of FF rather than IGF-1R-mediated pathways.

### 2.5. FF Increases Attached Growth of FE and HGSC Cells on Peritoneum, Partially Dependent on AKT

**Figure 5:**
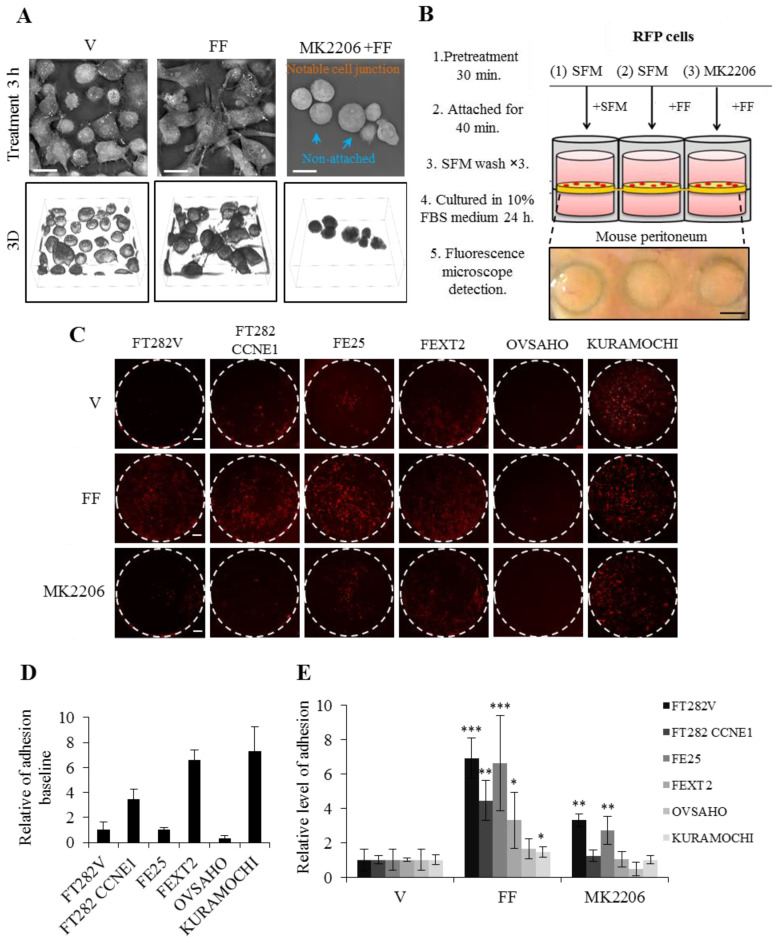
FF increases attachment growth of FTE cells on the peritoneum, partially dependent on AKT. (**A**) Label-free 3D refractive index image (Nanolive SA, Tolochenaz, Switzerland) of FE25 cells cultured in serum free, with or without 3h-FF treatment and AKT inhibition. (**B**) Schematic diagram of the ex vivo assay of attachment growth on peritoneum tissue as described in Materials and Methods. Scale bar, 1000 µm. (**C**) Representative image of adhesive growth of RFP-labelled cells on the mouse peritoneum with the same pretreatments. Scale bar, 200 μm. (**D**) Comparison of baseline peritoneal adhesion of different cells. By using Image J, adhered RFP+ cells were counted and compared using the value of FT282-V as a reference. (**E**) Changes of adhesion value after FF treatment with or without AKT inhibition (MK2206). The treat and no treat comparisons were done on the same peritoneum. Three or more independent experiments were done to get the mean value. Error bar represents mean ± SD, the asterisk represents comparison of vehicle treatment. * *p* < 0.05, ** *p* < 0.01, *** *p* < 0.001 by two-sided, unpaired Student’s *t*-test.

Upon treated with FF, we found a profound increase in cell attachment and projections on the culture dish. Figure 5A demonstrated this attachment and spreading in a three-dimensional refractive index imaging. After FF treatment for three hours in serum-free culture, the increase of cell spreading involved the organization of cytoskeleton with projections of lamellipodia and filopodia; pretreatment with AKT inhibitors could block this effect (Figure 5A). 

We further tested the effect of FF on the attachment growth on the peritoneum tissue cultured ex vivo. As shown in Figure 5B–E, the two transformed cell lines, FEXT2 and KURAMOCHI showed the highest adhesive growth, compared to almost no attachment in the other transformed cell OVSAHO. The poor attachment of OVSAHO corresponds to the xenograft feature, where peritoneal tumor seedings were in lesser volumes and were easily detached (Figure 5C–E and Figure 1G). FF markedly increased attachment of all the FTE cells (from 3.3- to 6.9-fold) and modestly increased attachment of the other transformed two cell lines (from 45% and 64%). AKT inhibition partially reduced this FF effect in all the cell lines (Figure 5E). The results indicated that FF markedly enhances attachment of transforming FTE cells and KURAMOCHI cells but not OVSAHO, and this is partly mediated by AKT signaling.

### 2.6. FF Highly Promotes the Motility of Transforming FE Cells, Partially Mediated by AKT

**Figure 6:**
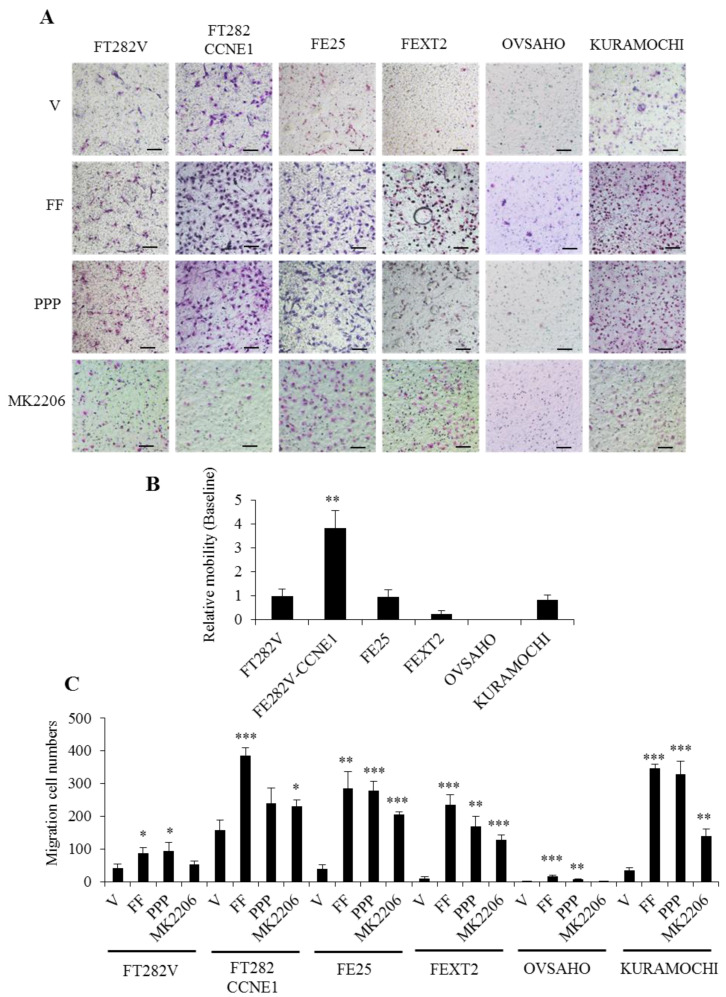
FF highly promotes the migration of FTE /HGSC cells, partially mediated by AKT. Under serum-free conditions, the transwell mobility assay was conducted using inserts with 8 μm pores and cultured in DMEM with 10% FF or vehicle as a chemoattractant in two layers of medium. The same PPP and MK2206 pretreatment was done for 30 min and detected at 18 h. (**A**) Representative images of migrated cells of each treatment group. Scale bar, 100 μm. (**B**) FT282-V cell line as a reference value to compare the baseline of cell motility. (**C**) The number of migrated cells per field were imaged and counted (mean ± SD; three independent experiments); * *p* < 0.05, ** *p* < 0.01, *** *p* < 0.001 by two-sided, unpaired Student’s *t*-test.

Figure 6A,B show the transwell migration of the cell lines. FT282-CCNE1 cell lines showed a high migration activity at baseline, which were 3.9-fold higher than the parental FT282-V cell line. The three transformed cells, especially the OVSAHO cell line that showed little migration, had relatively low migration activity. However, FF remarkably increased the motility of all FE and HGSC cells, up to 33.4-fold (OVSAHO cells) in serum-free culture conditions. The increase in motility could be positively related to the severity of transformation. Treatment with AKT inhibitor partly reduced this effect. Effects IGF-1R inhibition varied from partial to no response (Figure 6C). These results indicate that the enhanced motility effect of FF on FE and HGSC cells is partly mediated through the AKT signaling pathways.

### 2.7. FF Promotes the Invasion of Transforming FE Cells, Largely Dependent on AKT

**Figure 7:**
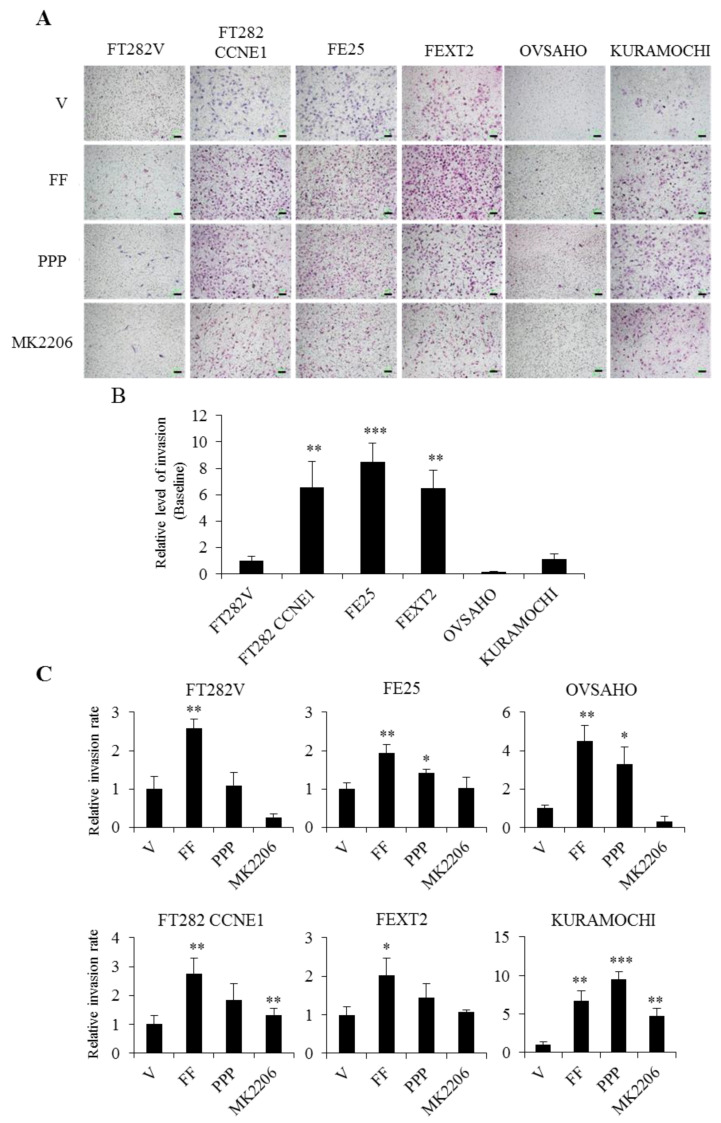
FF highly promotes the invasion of FTE /HGSC cells, largely by AKT signaling. Transwell invasion assay was conducted using matrigel coated insert with 8 μm pore size. Growth medium with 10% FF or vehicle served as a chemoattractant in two layers of medium. The same PPP and MK2206 pre-treatment were done for 30 min, and the number of migrated cells per field were imaged and counted at 48 h. (**A**) Representative images of invaded cells for each cell line. Scale bar, 100 μm. (**B**) Baseline value of cell invasion using FT282-V cell line as a reference. (**C**) Comparison of different treatment groups. Data were calculated with mean ± SD from three independent experiments; * *p* < 0.05, ** *p* < 0.01, *** *p* < 0.001 by two-sided, unpaired Student’s *t*-test.

The transwell matrigel invasion activity is shown in Figure 7A,B. Interestingly, the transforming FTE cells had a significant higher invasion activity than the two HGSC cells. OVSAHO showed minimal invasion activity, followed by KURAMOCHI and FT282-V. FF markedly increased the invasion of all FTE and HGSC cells (from 1.93 folds to 6.67-fold). This FF effect was totally reversed by AKT inhibition except in KURAMOCHI cells which showed partial revision (Figure 7C). PPP inhibition had a partial effect. Particularly, in FT282V and OVSAHO cells, the suppressions of FF-induced invasion by AKT inhibitor were over 200%, suggesting an endogeneous activation of AKT in these cell lines. The results indicate that an enhanced invasion effect of FF on FE and HGSC cells, is partly through IGF-1R signaling, and largely through AKT-mediated pathways.

### 2.8. FF Increase EMT in Transforming FTE Cells Independent of AKT

**Figure 8:**
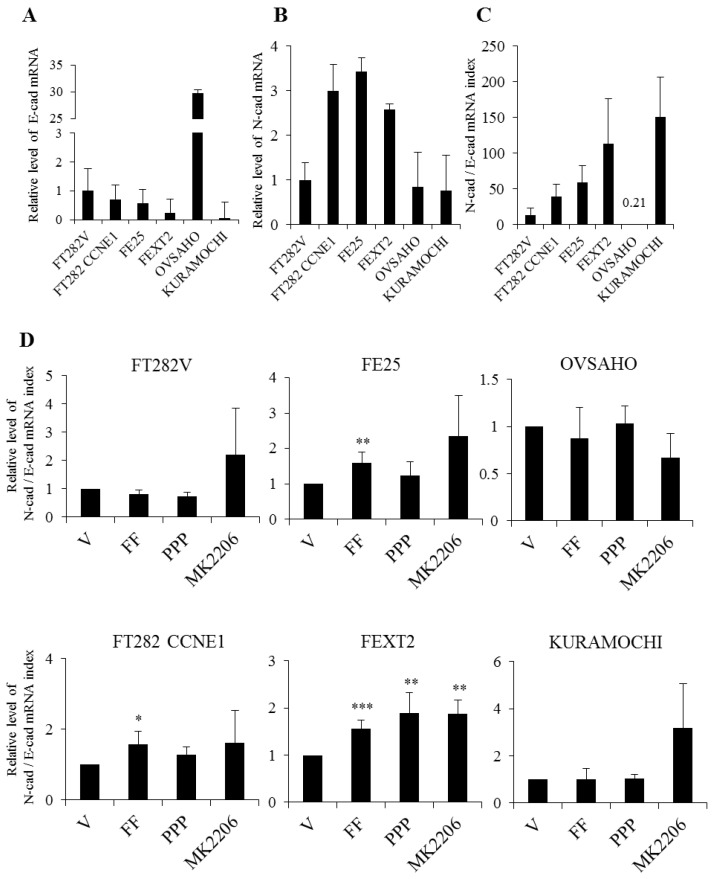
EMT assay of transforming FTE /HGSC cells. (**A**–**C**) mRNA levels of E-Cadherin and N-cadherin of each cell line were analyzed using real-time PCR. Baseline levels were shown with FT282-V as a reference. (**D**) EMT level, represented by N-cadherin/E-cadherin value, was compared in different cells with the same FF or vehicle treatment with or without IGF-1R or AKT inhibition. Mean ± SD from three or over three independent experiments. * *p* < 0.05, ** *p* < 0.01, *** *p* < 0.001 by two-sided, unpaired Student’s *t*-test.

Finally, we analyzed whether this migration- or invasion-enhancing activity of FF is through the induction EMT of cells. We detected mRNA level of E-cadherin (EC) as the epithelial cell marker and N-cadherin (NC) as the mesenchymal cell marker and used NC/EC ratio to represent the extent of EMT. As shown in Figure 8A–C, FTE transformation gradually increased the NC/EC ratio, however OVSAHO was an exception that showed an NC/EC ratio of 0.21, indicating an epithelial phenotype with least EMT. This corresponds with the low attachment, migration, and invasion phenotypes observed in vitro and in vivo. After treating with FF for 72 h, there was an increase of the NC/EC ratio in the three transforming FTE cells (FT282-CCNE1, FE25 and FEXT2) but not in HGSC cells. This increase was not altered by the two inhibitors (Figure 8D). 

## 3. Discussion

Fathalla first proposed the incessant ovulation theory of ovarian carcinogenesis [33]. Subsequently, Henderson et al. demonstrated the correlation between ovulation frequency and the incidence of ovarian cancer [34]. Over the past two decades, after the paradigm related to the origin of ovarian cancer has shifted from the ovarian surface to the tubal epithelium, several mechanistic evidences supporting the transformation activity of ovulation, specifically the carcinogens released from the ovulation follicle, are available [20,21,22,35,36,37]. Research focusing on the molecular mechanism of ovarian and peritoneal metastasis is also rising [38,39,40,41,42]. However, whether incessant ovulation also enhances peritoneal or ovarian metastasis is unknown. This study discovered that ovulatory FF promotes malignant transformation in all stages of ovarian cancer development from the FTE. 

Genetically engineered cell lines derived from the secretory cells of the fimbrial epithelium, the cell-of-origin of tubal carcinogenesis, and those derived from clinically overt HGSC all responded to aggravation of various transformation phenotypes. These phenotypes involve the early features of epithelial growth and transformation (proliferation, anoikis resistance, and AIG) and the later features of EMT and peritoneal metastasis (peritoneum attachment, migration, and invasion). As shown in Table 2, a transition from the epithelial to mesenchymal phenotype was observed during the transformation (FT282V vs. FT282-CCNE1) and peritoneal spreading (FE25 vs. FEXT2) of FTEC. This EMT is associated with a shift from a local growth and survival feature to a metastasis one of attachment and invasion at ectopic sites (except for the minimal transformed cell FT282V, which showed minimal growth). The same morphological and behavior dichotomy was also observed in the two HGSC cell lines where OVSAHO cells showed an epithelial phenotype and KUROMOCHI cells showed a mesenchymal phenotype. The observation is in line with the biology of epithelium transformation, where uncontrolled growth precedes the invasion and metastasis. This transition of differentiation seems to be actively happening in the advancement of HGSC and in response to chemotherapy [43]. 

Signaling through IGF-1R is largely responsible for AIG in all cell lines and cell migration and invasion in a part of the cell lines tested. Previously, we showed that IGF2 in FF, through the IGF-1R/AKT signaling, confers an AIG and tumorigenesis of transforming FTE cells [22]. Results of this study suggest that this transformation signal from FF is valid in all stages of HGSC development and for the later phenotypes of transformation. As summarized in Table 3, all the tested phenotypes inducible by FF are largely (AIG, proliferation, invasion) or partly (anoikis resistance, peritoneal attach, migration) mediated by AKT. AKT-kinases mediate a variety of signaling pathways in response to growth and environmental signals to regulate diverse cellular activities including cell proliferation and survival, tissue invasion, response to nutrients, and angiogenesis [44]. A plethora of evidences has indicated that AKT activation is one of the most common molecular alterations in cancer [45]. This study revealed a progressive increase in AKT phosphorylation along the severity of FTE cell transformation, and FF enhanced this effect in all cell lines tested. Autophosphorylation of IGF-1R/AKT was observed in the two fully transformed cell lines, which was further enhanced by FF. This pattern of activation of AKT and enhancement by FF correlated well with the levels of AIG of the cell panel. Thus, AKT signaling is one of the most important transforming niche of HGSC and serves as a key target for prevention and treatment [46,47].

Normally, cells rely on integrins to sense and interact with the extracellular matrix (ECM) to maintain a healthy state [48]. When detached from the stroma, cells undergo anoikis and this connection is lost [49]. A metastasizing cancer cell escapes this requirement of integrin–ECM interactions by hyper activating survival and proliferative cascades via the receptor tyrosine kinases [49]. We found that FF enhanced survival to anoikis, particularly in the highly transformed cells. This anoikis resistance activity as well as the mitogenic activity of FF was not conferred by IGF-1R and was largely or partially contributed by AKT. Other growth factors in FF, such as platelet-derived growth factor (PDGF) [36], hepatocyte growth factor (HGF) [50] and fibroblast growth factor-basic (bFGF) [51], may transmit these proliferative and survival signals.

In the proposed model of intraperitoneal seeding of ovarian cancer cells [52,53], cells were detached from the tubal epithelium, clustered into multicellular aggregates and adhered to the peritoneum for growth. We developed ex vivo tests to analyze the effect of FF on the peritoneal attachment of different cell lines. The results showed that FF vastly enhances attachment growth of cells with less baseline EMT and attachment activity. These are transforming FE cells such as in FT282V, FT282-CCNE1, and FE25. In contrast, the transformed FEXT2 cell and KURAMOCHI cells, which showed high EMT indexes and attachment growth, showed smaller but significant magnetite of increase of peritoneal attachment by FF (Table 2). The results indicate FF could enhance the peritoneal spreading of FTE cells at different stages of transformation. In a mouse model of ovarian tumor initiating cells, superovulation enhanced ovarian metastasis and stromal cell-derived factor 1 (SDF-1) expressed by granulosa cells showed chemo attraction activity [54]. The exact factors in FF that confer the attachment and growth of transforming and transformed FTE cells remain to be identified. 

The greatest impact of FF on FTE and HGSC cell lines is the enhancement of cell migration, by tens folds in the highly transformed FTEC cells and HGSC cells. Enhanced cell motility reflects the activity of detaching from cell-cell and cell-basement membrane adhesion and indicates a higher activity in spreading and invasion after attachment to the peritoneum or the ovarian surface. For this enhancement, different cells showed different dependency on IGF-1R and AKT. IGF-1R/AKT is partly responsible for the increase in OVSAHO and FEXT2 and FT282-CCNE1 cells, but in KURAMOCHI cells, non-IGF-R-mediated AKT was partly responsible. 

The migration-, invasion-, and adhesion-promoting activity of ovulatory FF may be fundamentally important for ovulation. The release of the oocyte from the ovarian follicle requires highly controlled degradation of the ovarian wall to allow passage of the oocyte and accompanying cells in the cumulus oocyte complex (COC) from the follicle. Evidences showed that COC acquires transient migratory, matrix-invading and adhesive capacities at ovulation. The migration activity reaches a peak at the time of ovulation or 12 h post-hCG induction, when COC cells quickly adhere to the extracellular matrix proteins present in the ovarian wall, and the invasion activity through the matrigel barrier reach a level as high as that of a breast cancer cell line [55].

This study identified a large part of the later transforming phenotypes including anoikis resistance, attachment growth, cell migration and cell invasion are not mediated by AKT. The nature of these activities is unknown. Promising candidates of non-AKT signals include soluble ECM proteins, such as laminin, fibronectin in FF [56] in promoting the adhesion, spreading, and migration of tumor cells via FAK [16,57,58,59]. 

We specially chose KURAMOCHI and OVSAHO cell lines to represent the Brcaness type (with BRCA2 mutation) and the non-Brcaness (with null-Rb and CCNE amplification) HGSC, respectively [4,5]. These two cell types showed different basal characteristics of the transformation, FF responsiveness, and IGF-1R and AKT dependence. Compared to the KUROMACHI cell, the non-Brcaness OVSAHO cell showed a higher basal level and FF-responsiveness in AIG and anoikis resistance, primarily dependent on IGF-1R and AKT signaling. This cell showed an epithelioid phenotype with a low EMT index and poor peritoneal attachment, migration, and invasion (Table 2). In contrast, the Brcaness KUROMACHI cell showed mesenchymal phenotypes of high EMT index and the attachment, migration, and invasion activities that are further enhanced by FF. KUROMACHI cell is also characterized by a low AIG and low anoikis resistance, and a higher proliferation. FF enhanced all these activities as well. Thus regardless of the genotypes regarding the homologous recombination repair genes, FF enhances the full spectrum of phenotypes in the transformation and peritoneal metastasis of HGSC.

One concern of the significance of this study is that HGSCs are commonly found in menopausal women with ceased ovulation. The median age at diagnosis for ovarian HGSC was 57 years [60]. According to the SEER report (https://ocrahope.org/patients/about-ovarian-cancer/statistics/), 17.1% of ovarian cancer was diagnosed at age 45–54, and 10.5% was diagnosed at age 20–45. Moreover, the transformation of tubal epithelium likely initiates at p53 signature, and it is as early as in the STIC stage that peritoneal spreading starts [14]. According to an estimation from epidemiological and molecular pieces of evidence, the natural history of the step-wise development of HGSC spans for more than 30 years starting from the menarche, taking ten years from normal epithelium to p53 signature, 15 years from p53 signature to STIC, and five years from STIC to HGSC [11,12]. The majority of the developmental course of HGSC is in the ovulatory age.

## 4. Material and Methods

### 4.1. Clinical Specimens

FF aspirates were collected from subjects undergoing oocyte retrieval in an IVF program as previously described [20,21,22]. Fresh FFs without blood contamination were cryopreserved in batches. Among them, 50 FFs were pooled, aliquoted, and used for subsequent experiments. Each aliquot was frozen and thawed for a maximum of two times before experiments. This study involves two research programs (TCRD-I102–01-01 and MOST 107-2314-B-303-013-MY3), which were approved by the institutional review board of Tzu Chi Medical Center, Taiwan (Approval No. IRB-101-09, IRB -106-07-A).

### 4.2. Human Fallopian Tube Fimbrial Epithelial Cell Lines

#### Cell Sources

Three immortalized human fimbrial epithelial cell lines (FT282-V, FT282-cyclin E1 (CCNE1), FE25), one subline derived from the xenograft tumor of FE25 cells (FEXT2), and two genomically-proven HGSC cell lines (OVSAHO and KURAMOCHI) were used in this study. The relevant genotypes and phenotypes are shown in Table 1. We established FE25 cells by transduction using human papillomavirus (HPV) E6/E7 plus human telomerase reverse transcriptase (hTERT) [30]. FT282-CCNE1 and FT282-V cell lines, a kind gift from Dr. Ronny Drapkin, were transduced with *TP53* p.R175H, *hTERT* plus *CCNE1* or vector, respectively [23]. These cells were maintained in MCDB105/M199 medium (1:1, Merck, NJ, USA) supplemented with 10% fetal bovine serum (FBS, Thermo Fisher Scientific, Waltham, MA, USA) and penicillin/streptomycin (P/S, Corning Inc., Corning, NY, USA). The HGSC cell lines, KURAMOCHI and OVSAHO were obtained from the JCRB cell bank, Japan. The cells were cultured in RPMI 1640 medium (Gibco-Thermo Fisher Scientific, MA, USA) supplemented with 10% (*v*/*v*) FBS, penicillin (50 U/mL) and streptomycin (50 μg/mL).

### 4.3. Xenograft Tumor Model

To analyze the tumorigenicity of the cell lines used in the study, we generated NOD/Shi-scid/IL-2Rγnull (NSG) xenograft mice models (Jackson Laboratory, Bar Harbor, ME, USA). Briefly, 8-week-old mice (5 to 12 per group) were injected intraperitoneally with FE cells in 200 µL of PBS or 10% FF plus 10% matrigel (Corning Inc.). PBS or 10% FF were boosted once or twice a week for six weeks. Animals were sacrificed at the end of six months (FE25 cell), the seventh week (OVSAHO-RFP cell) or after four and a half months (KURAMOCHI cell), or when tumor burden was evident or general health was determined to be moribund. The in vivo imaging systems (IVIS^®^, PerkinElmer, Shelton, CT, USA) was used to observe tumors in OVSAHO-RFP xenografts. Post-image processing and quantification were performed using Image J software (Rasband, W.S., ImageJ, U. S. National Institutes of Health, Bethesda, MD, USA, https://imagej.nih.gov/ij/, 1997–2018), and the intensity scales were normalized and the region of interest (ROI) was measured. Calculation of the overall signal of red fluorescence was performed with saturation between 50 and 255. All experimental procedures involving mice were conducted under the approved guidelines of the Animal Care and Use Committee of Tzu-Chi University (Approval ID: 108–25).

### 4.4. Immunohistochemistry Analysis

Tissue sections were deparaffinized and treated with 0.3% hydrogen peroxide to block endogenous peroxidase activity. Antigens were retrieved from the tissues by incubation with appropriate antigen retrieval buffer in an oven for 45 min followed by staining with primary antibodies at 4 °C overnight. Antibodies used in this study are listed in Table S2. Sections were stained using UltraVision™ Quanto Detection System (Thermo Fisher) and imaged using Zeiss microscope (Axio Vert A1, Oberkochen, Germany).

### 4.5. Western Blot Analysis

Briefly, 40 μg of total sample protein was separated on a 10% sodium dodecyl sulfate polyacrylamide gel electrophoresis (SDS-PAGE). After electrophoresis, gels were transferred to immobilon-P membranes (EMD Millipore, Burlington, MA, USA). Polyvinylidene fluoride (PVDF) membranes were blocked using 5% (*w/v*) non-fat milk powder in phosphate-buffered saline (PBS) with 0.1% Tween-20 (PBST) at room temperature (RT) for one hour and incubated with primary antibody at 4 °C overnight. After a PBST wash, blots were incubated with secondary antibody in PBST at RT for one hour. The blots were exposed to chemiluminescent horse radish peroxidase (HRP) antibody detection reagent (EMD Millipore, MA, USA). The quantitative analysis of the protein of interest was performed using Image J software. All antibodies used in the study are listed in Table S2.

### 4.6. AIG

The AIG assay was modified for this study using the 96-well plate. Briefly, aliquots of agarose (Invitrogen, Carlsbad, CA, USA) (2% *w*/*v* in water) were sterilized and stored at RT in 50 mL tubes. The agar was melted and kept in a water bath at 41 °C. The bottom layer (0.8% soft agar) was prepared with MCDB/M199 in 10% FBS growth medium. The top layer (0.4% soft agar), was prepared with 2000 cells/well with or without 10% FBS MCDB/M199 medium. After 14 days, number of colonies was randomly counted at 100× magnification.

### 4.7. Anoikis Resistance Assay

For anoikis resistance assay, modified protocol of CytoSelectTM 96-Well Anoikis Assay (Cat# CBA-081, Cell Biolabs Inc., San Diego, CA, USA) was followed. Briefly, after pretreatment with IGF-1R inhibitor (1 nM PPP), AKT inhibitor (10 µM MK2206) or vehicle for 30 min, cells were inoculated into agarose coated 96-well plates with 2 × 10^3^ cells/well in serum-free medium. The cells were treated with or without FF (10%) once in 72 h, and then cell viability was determined using the XTT colorimetric assay for 24 h. The cell-free background values were subtracted. 

### 4.8. Ex Vivo Peritoneal Attachment Growth Assay

The panel cells were transduced with red fluorescent protein (RFP) TRITC lentivirus (pLAS2w.RFP-C.Ppuro, from Taiwan RNAi core facility) for fluorescence detection. For the ex vivo adhesion assay, a 3 cm × 2 cm size peritoneum sheet was dissected from a female C57BL/6 mice. After a PBS wash for 30 min, the peritoneum membrane was placed as an insert in the 48-well chemotaxis chamber (Neuro Probe; Cabin John, MD, USA) (Figure 5B). On the serosa surface of the peritoneum insert, we loaded 2000 RFP-labeled cells with the same PPP/MK2206 inhibitor- or vehicle-pretreatment, and with or without 10% FF. The upper and lower chambers were filled with serum-free culture medium. After 40 min, the surface was gently washed three times with serum-free medium and cultured for 24 h with normal medium with 10% FBS. Then RFP-positive cell colony on the insert was counted using Image J software. 

### 4.9. Cell Motility and Invasion Assay

The cell motility assays were performed using a 24-well transwell chamber system (Costar 3422, Corning Inc.). Cells were seeded in the upper chamber at 2 × 10^4^ cells in 0.3 mL serum-free MCDB/M199 media. After incubation for 18 h at 37 °C in an atmosphere containing 5% CO_2_, media supplemented with 10% FF was placed in the upper and lower wells in a total volume of 0.5 mL. The membranes were fixed in 4% paraformaldehyde for 20 min. Migrated cells on the lower surface were stained with Giemsa. For invasion assay, transwell membranes were pre-coated with 60 μL diluted matrix matrigel (Corning Inc.) overnight. To the upper chamber, 1 × 10^4^ cells were loaded. Cells that migrated to the lower chamber were counted after 48 h at three random fields per filter.

### 4.10. Epithelial to Mesenchymal Transition (EMT) Assay

Real-time polymerase chain reaction (PCR) was used to examine the mRNA expression levels of E-cadherin (EC) and N-cadherin (NC) at the mRNA level. PPP (1 nM) and MK2206 (10 µM) cell groups were pre-treated with the respective inhibitors for 30 min in serum-free conditions, and then 10% FF was added and incubated for 72 h. Total RNA was extracted from untreated or treated cells using the GeneJET RNA Purification Kit (Thermo Fisher Scientific) according to the manufacturer’s instructions. One microgram of RNA was reverse transcribed to complementary DNA using RevertAid First Strand cDNA Synthesis Kit (Thermo Fisher Scientific). Real-time PCR was carried out at 95°C, 2 min; 40× (95 °C, 10 s; 55 °C, 30 s), using SYBR Premix Ex Taq (Takara Biotechnology Co., Ltd., Dalian city, China) and primers (listed in Table S2). The expression level of each target gene was normalized to that of β-actin. Relative mRNA expression levels were determined using the 2−∆∆Ct method. The EMT index was calculated using the formula: N-cad = 2^(−∆∆CT)^ /E-cad = 2^(−∆∆CT)^. The statistical analysis was performed based on the ratio of three or more independent experiments.

### 4.11. Statistics

All data are presented as mean ± standard error. Statistical analyses were performed using GraphPad Prism version 8.0 (GraphPad Software, La Jolla, CA, USA) and Microsoft Office Excel 2010 (Microsoft, Redmond, WA, USA). Detailed information on statistical analysis is described in figure legends.

## 5. Conclusions

Taken together, the study indicates that through the spectrum of carcinogenic activities of follicular fluid, ovulation may enhance malignant transformation of fallopian tube epithelial cells in the full journey of the development of ovarian HGSC. This on one hand would reinforce ovulation break, either by pregnancy/lactation or by oral contraceptives, as the most powerful strategy for prevention of ovarian cancer. On the other hand, given that FF also aggravates malignancy of established HGSC cells, ovulation inhibition and precision fertility management is important for early stage I ovarian cancer patients who have undertaken a fertility sparing treatment to preserve healthy ovary at surgery.

## Figures and Tables

**Table 1:** Immortalized human fimbrial epithelial cell lines and HGSC cell lines.

Cell Line	Origin	Genetic Alterations	Mimic	AIG Colony (>50 μm)/AVG.	Tumorigenesis in NSG Mice
FT282V [30]	Normal fimbrial epithelium	hTERT + TP53 p.R175H	p53 signature	0	0
FT282-CCNE [30]	FT282	hTERT + TP53 p.R157H + CCNE1	STIC	0.33	2/8 (25%)
FE25 (p110)	Normal fimbrial epithelium	hTERT + HPV E6/E7 (p53/Rb loss)	STIC	2	0
FEXT2	ip xenotumor from FE25 + FF	hTERT + HPV E6/E7 (p53/Rb loss)	Perit. STIC	1.2	5/5 (100%)
OVSAHO	Abdominal metastasis of HGSC	TP53 p.R342* (Nonsense), Rb-null, NF1 mut; CCNE, FGFR4 amp, BRCA2 homodel; MAP2K4 hetloss. [28,29]	HGSC	1.6	4/4 (100%)
KURAMOCHI	Ascites of HGSC	TP53 p.D281Y, BRCA2 mutation; NF1 homodel; KRAS, MYC, FGFR1, CCNE, ARID1A amp. [28,29]	HGSC (BRCAness)	0	5/5 (100%)

**Table 2:** Transition of the epithelial (E) and mesenchymal (M) phenotypes in FTE transformation and in HGSC.

Cell Line	Mimic	Phenotype Summary *	Growth and Transformation		Peritoneal Metastasis			
			AIG **	Proliferation	EMT (N/E-CAD)	Peritoneal Attachment	Migration	Invasion
FT282V	p53 signature	E	0	0.3	13	72	41	25
FT282-CCNE1	STIC	M	0.3	1.9	40	251	159	162
FE25	STIC	EM	2.0	1.7	59	74	40	210
FEXT2	Perit. STIC	M	1.2	0.9	114	474	10	159
OVSAHO	HGSC	E	1.6	0.3	0.21	24	0.5	3
KURAMOCHI	HGSC	M	0	1.2	151	526	35	27

* E: Epithelium-like, M: Mesenchyma-ike, EM: mixed populations of E and M; ** Definitions of scales of transformation phenotypes at baseline are AIG: colony (>50 μm or >25 μm) number in soft agar; Proliferation: Value in XTT assay; Anoikis resistance: Value of XTT assay in non-attached culture; Attachment growth: Fluorescence level of cells attached to human mesothelial cells; Cell mobility: Number of cells migrated to the lower part of transwell; Invasion: Number of cell in the lower part of transwell with matrigel insert; EMT: mRNA ratio of N-cadherin/E-cadhern.

**Table 3:** Effect of FF and FF + kinase inhibitors in transformation phenotypes of FE and HGSC cells.

Cell Line	AIG **			Proliferation **			Anoikis Resistance **			Peritoneal Attachment **			Migration **			Invasion **			EMT (N-Cad/E-Cad) **		
	+FF	+PPP	+MK	+FF	+PPP	+MK	+FF	+PPP	+MK	+FF	+PPP	+MK	+FF	+PPP	+MK	+FF	+PPP	+MK	+FF	+PPP	+MK
FT282V	0.5 (>25μm)	0.17 (>25μm)	0	▴▴	▴	▴	▴▴▴▴	▴▴▴▴	▴▴▴	6.9×	N.D.	3.31×	2.2×	2.3×	1.3×	2.6×	1.1×	0.3×	✧	✧	✧
FT282-CCNE1	19.2×	7.1×	3.0×	▴▴	▴▴	▴	▴▴▴▴	▴▴▴▴	▴▴▴	4.5×	N.D.	1.23×	2.4×	1.5×	1.4×	2.8×	1.8×	1.3×	▴▴▴	✧	✧
FE25	4.7×	1.3×	0.7×	▴	▴▴	▴	▴▴▴	▴▴▴	▴	6.6×	N.D.	2.72×	2.0×	7.0×	5.2×	1.9×	1.4×	1.0×	▴▴▴	✧	✧
FEXT2	7.4×	3.6×	0.3×	▴▴	▴▴	▴	▴▴▴▴	▴▴▴	▴▴▴	3.3×	N.D.	1.03×	24.0×	17×	13×	2.0×	1.3×	1.1×	▴▴▴	▴▴▴	▴▴▴
OVSAHO	10.0×	3.4×	1.7×	▴▴▴	▴▴	▴	▴▴▴▴	▴▴▴▴	▴▴▴	1.6×	N.D.	0.47×	33.4×	11.6×	0.0×	4.5×	3.3×	0.3×	✧	✧	✧
KURAMOCHI	3.0 (>25μm)	0	0.7 (>25μm)	▴▴▴	▴▴▴	▴▴	▴▴▴	▴▴▴	▴▴▴	1.5×	N.D.	1.00×	9.9×	9.4×	4.0×	6.7×	9.5×	4.8×	✧	✧	✧
Role of IGF/AKT in FF	IGF and AKT dependent			IGF independent AKT largely dependent			IGF independentAKT partially dependent			IGF independent AKT partially dependent			IGF and AKT partially dependent			IGF independent AKT largely dependent			Nil		

Definitions of scales of transformation phenotypes at baseline are AIG: colony (>50 μm or >25 μm when indicated) number in soft agar; Proliferation: Value in XTT assay; Anoikis resistance: Value of XTT assay in non-attached culture; Attachment growth: Fluorescence level of cells attached to human mesothelial cells; Cell mobility: Number of cells migrated to the lower part of transwell; Invasion: Number of cell in the lower part of transwell with matrigel insert; EMT: mRNA ratio of N-cadherin/E-cadherin. ** Changes after FF treatment with or without inhibitors were scaled as the following: (1) For AIG, absolute AIG colony number (>25 μm) was showed directly in cells with a baseline level of zero. Folds of change from baseline level were showed in other cells. (2) For peritoneal attachment, migration and invasion, folds of change from baseline level were showe. (3) For other phenotypes with less prominent alterations, changes were symbolized as ✧: No significant change, ▴: 0–20% increase, ▴▴: 20–50% increase, ▴▴▴: 50–100% increase, ▴▴▴▴: >100% increase.

## Data Availability

The data presented in this study are available in this article (and supplementary material).

## Supplementary Materials

The following are available online at https://www.mdpi.com/2072-6694/13/3/468/s1, Figure S1: Gross and histochemical characteristics of xenograft tumors of OVSAHO-RFP cells, Figure S2: Full Western Blot images for Figure 2A, Table S1: Continuous stimulation of xenograft incidence and tumor site by follicular fluid, Table S2: Primary antibodies for WB and IHC.

## References

[1] Coleman M.P., Forman D., Bryant H., Butler J., Rachet B., Maringe C., Nur U., Tracey E., Coory M., Hatcher J. (2011). Cancer survival in Australia, Canada, Denmark, Norway, Sweden, and the UK, 1995–2007 (the International Cancer Benchmarking Partnership): An analysis of population-based cancer registry data. Lancet.

[2] Dao F., Schlappe B.A., Tseng J., Lester J., Nick A.M., Lutgendorf S.K., McMeekin S., Coleman R.L., Moore K.N., Karlan B.Y. (2016). Characteristics of 10-year survivors of high-grade serous ovarian carcinoma. Gynecol. Oncol..

[3] Levanon K., Crum C., Drapkin R. (2008). New insights into the pathogenesis of serous ovarian cancer and its clinical impact. J. Clin. Oncol..

[4] Vang R., Shih I.M., Kurman R.J. (2013). Fallopian tube precursors of ovarian low- and high-grade serous neoplasms. Histopathology.

[5] Ducie J., Dao F., Considine M., Olvera N., Shaw P.A., Kurman R.J., Shih I.M., Soslow R.A., Cope L., Levine D.A. (2017). Molecular analysis of high-grade serous ovarian carcinoma with and without associated serous tubal intra-epithelial carcinoma. Nat. Commun..

[6] Leeper K., Garcia R., Swisher E., Goff B., Greer B., Paley P. (2002). Pathologic findings in prophylactic oophorectomy specimens in high-risk women. Gynecol. Oncol..

[7] Folkins A.K., Jarboe E.A., Saleemuddin A., Lee Y., Callahan M.J., Drapkin R., Garber J.E., Muto M.G., Tworoger S., Crum C.P. (2008). A candidate precursor to pelvic serous cancer (p53 signature) and its prevalence in ovaries and fallopian tubes from women with BRCA mutations. Gynecol. Oncol..

[8] Kindelberger D.W., Lee Y., Miron A., Hirsch M.S., Feltmate C., Medeiros F., Callahan M.J., Garner E.O., Gordon R.W., Birch C. (2007). Intraepithelial carcinoma of the fimbria and pelvic serous carcinoma: Evidence for a causal relationship. Am. J. Surg. Pathol..

[9] Kuhn E., Kurman R.J., Vang R., Sehdev A.S., Han G., Soslow R., Wang T.L., Shih I.M. (2012). TP53 mutations in serous tubal intraepithelial carcinoma and concurrent pelvic high-grade serous carcinoma—Evidence supporting the clonal relationship of the two lesions. J. Pathol..

[10] Labidi-Galy S.I., Papp E., Hallberg D., Niknafs N., Adleff V., Noe M., Bhattacharya R., Novak M., Jones S., Phallen J. (2017). High grade serous ovarian carcinomas originate in the fallopian tube. Nat. Commun..

[11] Wu R.C., Wang P., Lin S.F., Zhang M., Song Q., Chu T., Wang B.G., Kurman R.J., Vang R., Kinzler K. (2019). Genomic landscape and evolutionary trajectories of ovarian cancer precursor lesions. J. Pathol..

[12] Wu N.Y., Fang C., Huang H.S., Wang J., Chu T.Y. (2020). Natural history of ovarian high-grade serous carcinoma from time effects of ovulation inhibition and progesterone clearance of p53-defective lesions. Mod. Pathol..

[13] Jarboe E., Folkins A., Nucci M.R., Kindelberger D., Drapkin R., Miron A., Lee Y., Crum C.P. (2008). Serous carcinogenesis in the fallopian tube: A descriptive classification. Int. J. Gynecol. Pathol..

[14] Bijron J.G., Seldenrijk C.A., Zweemer R.P., Lange J.G., Verheijen R.H., van Diest P.J. (2013). Fallopian tube intraluminal tumor spread from noninvasive precursor lesions: A novel metastatic route in early pelvic carcinogenesis. Am. J. Surg. Pathol..

[15] Wang K.H., Chu S.C., Chu T.Y. (2017). Loss of calponin h1 confers anoikis resistance and tumor progression in the development of high-grade serous carcinoma originating from the fallopian tube epithelium. Oncotarget.

[16] Farsinejad S., Cattabiani T., Muranen T., Iwanicki M. (2019). Ovarian Cancer Dissemination-A Cell Biologist’s Perspective. Cancers.

[17] Beral V., Doll R., Hermon C., Peto R., Reeves G., Collaborative Group on Epidemiological Studies of Ovarian Cancers (2008). Ovarian cancer and oral contraceptives: Collaborative reanalysis of data from 45 epidemiological studies including 23,257 women with ovarian cancer and 87,303 controls. Lancet.

[18] Holschneider C.H., Berek J.S. (2000). Ovarian cancer: Epidemiology, biology, and prognostic factors. Semin. Surg. Oncol..

[19] Havrilesky L.J., Moorman P.G., Lowery W.J., Gierisch J.M., Coeytaux R.R., Urrutia R.P., Dinan M., McBroom A.J., Hasselblad V., Sanders G.D. (2013). Oral contraceptive pills as primary prevention for ovarian cancer: A systematic review and meta-analysis. Obstet. Gynecol..

[20] Huang H.S., Chu S.C., Hsu C.F., Chen P.C., Ding D.C., Chang M.Y., Chu T.Y. (2015). Mutagenic, surviving and tumorigenic effects of follicular fluid in the context of p53 loss: Initiation of fimbria carcinogenesis. Carcinogenesis.

[21] Huang H.S., Hsu C.F., Chu S.C., Chen P.C., Ding D.C., Chang M.Y., Chu T.Y. (2016). Haemoglobin in pelvic fluid rescues Fallopian tube epithelial cells from reactive oxygen species stress and apoptosis. J. Pathol..

[22] Hsu C.F., Huang H.S., Chen P.C., Ding D.C., Chu T.Y. (2019). IGF-axis confers transformation and regeneration of fallopian tube fimbria epithelium upon ovulation. EBioMedicine.

[23] Karst A.M., Jones P.M., Vena N., Ligon A.H., Liu J.F., Hirsch M.S., Etemadmoghadam D., Bowtell D.D., Drapkin R. (2014). Cyclin E1 deregulation occurs early in secretory cell transformation to promote formation of fallopian tube-derived high-grade serous ovarian cancers. Cancer Res..

[24] Kuhn E., Wang T.L., Doberstein K., Bahadirli-Talbott A., Ayhan A., Sehdev A.S., Drapkin R., Kurman R.J., Shih I.M. (2016). CCNE1 amplification and centrosome number abnormality in serous tubal intraepithelial carcinoma: Further evidence supporting its role as a precursor of ovarian high-grade serous carcinoma. Mod. Pathol..

[25] Yim E.K., Park J.S. (2005). The role of HPV E6 and E7 oncoproteins in HPV-associated cervical carcinogenesis. Cancer Res. Treat..

[26] Sen P., Ganguly P., Ganguly N. (2018). Modulation of DNA methylation by human papillomavirus E6 and E7 oncoproteins in cervical cancer. Oncol. Lett..

[27] Domcke S., Sinha R., Levine D.A., Sander C., Schultz N. (2013). Evaluating cell lines as tumour models by comparison of genomic profiles. Nat. Commun..

[28] The Cancer Genome Atlas Research Network (2011). Integrated genomic analyses of ovarian carcinoma. Nature.

[29] Papp E., Hallberg D., Konecny G.E., Bruhm D.C., Adleff V., Noe M., Kagiampakis I., Palsgrove D., Conklin D., Kinose Y. (2018). Integrated Genomic, Epigenomic, and Expression Analyses of Ovarian Cancer Cell Lines. Cell Rep..

[30] Shin H.Y., Yang W., Lee E.J., Han G.H., Cho H., Chay D.B., Kim J.H. (2018). Establishment of five immortalized human ovarian surface epithelial cell lines via SV40 T antigen or HPV E6/E7 expression. PLoS ONE.

[31] Desmeules P., Trudel D., Turcotte S., Sirois J., Plante M., Gregoire J., Renaud M.C., Orain M., Tetu B., Bairati I. (2015). Prognostic significance of TIMP-2, MMP-2, and MMP-9 on high-grade serous ovarian carcinoma using digital image analysis. Hum. Pathol..

[32] Elias K.M., Emori M.M., Papp E., MacDuffie E., Konecny G.E., Velculescu V.E., Drapkin R. (2015). Beyond genomics: Critical evaluation of cell line utility for ovarian cancer research. Gynecol. Oncol..

[33] Fathalla M.F. (1971). Incessant ovulation—A factor in ovarian neoplasia?. Lancet.

[34] Casagrande J.T., Louie E.W., Pike M.C., Roy S., Ross R.K., Henderson B.E. (1979). ”Incessant ovulation” and ovarian cancer. Lancet.

[35] Lin S.F., Gerry E., Shih I.M. (2017). Tubal origin of ovarian cancer—The double-edged sword of haemoglobin. J. Pathol..

[36] Yeh C.H., Chen P.C., Chen C.H., Hsu C.F., Huang R.L., Ding D.C., Chu T.Y. (2016). Platelet-Derived Growth Factor in the Ovarian Follicle Attracts the Stromal Cells of the Fallopian Tube Fimbriae. PLoS ONE.

[37] King S.M., Hilliard T.S., Wu L.Y., Jaffe R.C., Fazleabas A.T., Burdette J.E. (2011). The impact of ovulation on fallopian tube epithelial cells: Evaluating three hypotheses connecting ovulation and serous ovarian cancer. Endocr. Relat. Cancer.

[38] Allen B.J., Brown J.K., Mountford M.H., Tamat S.R., Patwardhan A., Moore D.E., Ichihashi M., Mishima Y., Kahl S.B. (1989). In vitro and in vivo studies of boron conjugated melanoma affined biochemicals. Strahlenther. Onkol..

[39] Roggiani F., Mezzanzanica D., Rea K., Tomassetti A. (2016). Guidance of Signaling Activations by Cadherins and Integrins in Epithelial Ovarian Cancer Cells. Int. J. Mol. Sci.

[40] Mikula-Pietrasik J., Uruski P., Tykarski A., Ksiazek K. (2018). The peritoneal “soil” for a cancerous “seed”: A comprehensive review of the pathogenesis of intraperitoneal cancer metastases. Cell. Mol. Life Sci..

[41] Motohara T., Masuda K., Morotti M., Zheng Y., El-Sahhar S., Chong K.Y., Wietek N., Alsaadi A., Karaminejadranjbar M., Hu Z. (2019). An evolving story of the metastatic voyage of ovarian cancer cells: Cellular and molecular orchestration of the adipose-rich metastatic microenvironment. Oncogene.

[42] Coelho R., Ricardo S., Amaral A.L., Huang Y.L., Nunes M., Neves J.P., Mendes N., Lopez M.N., Bartosch C., Ferreira V. (2020). Regulation of invasion and peritoneal dissemination of ovarian cancer by mesothelin manipulation. Oncogenesis.

[43] Chen X., Zhang J., Zhang Z., Li H., Cheng W., Liu J. (2013). Cancer stem cells, epithelial-mesenchymal transition, and drug resistance in high-grade ovarian serous carcinoma. Hum. Pathol..

[44] Altomare D.A., Testa J.R. (2005). Perturbations of the AKT signaling pathway in human cancer. Oncogene.

[45] Bellacosa A., Kumar C.C., Di Cristofano A., Testa J.R. (2005). Activation of AKT kinases in cancer: Implications for therapeutic targeting. Adv. Cancer Res..

[46] Lengyel C.G., Altuna S.C., Habeeb B.S., Trapani D., Khan S.Z. (2020). The Potential of PI3K/AKT/mTOR Signaling as a Druggable Target for Endometrial and Ovarian Carcinomas. Curr. Drug Targets.

[47] Shariati M., Meric-Bernstam F. (2019). Targeting AKT for cancer therapy. Expert Opin. Investig. Drugs.

[48] Guadamillas M.C., Cerezo A., Del Pozo M.A. (2011). Overcoming anoikis—Pathways to anchorage-independent growth in cancer. J. Cell Sci..

[49] Frisch S.M., Ruoslahti E. (1997). Integrins and anoikis. Curr. Opin. Cell Biol..

[50] Sahin N., Toylu A., Gulekli B., Dogan E., Kovali M., Atabey N. (2013). The levels of hepatocyte growth factor in serum and follicular fluid and the expression of c-Met in granulosa cells in patients with polycystic ovary syndrome. Fertil. Steril..

[51] Seli E., Zeyneloglu H.B., Senturk L.M., Bahtiyar O.M., Olive D.L., Arici A. (1998). Basic fibroblast growth factor: Peritoneal and follicular fluid levels and its effect on early embryonic development. Fertil. Steril..

[52] Sun F., Feng M., Guan W. (2017). Mechanisms of peritoneal dissemination in gastric cancer. Oncol. Lett..

[53] Roque R., Costa Sousa F., Figueiredo-Dias M. (2020). Epithelial-mesenchymal interconversions in ovarian cancer: The levels and functions of E-cadherin in intraabdominal dissemination. Oncol. Rev..

[54] Yang-Hartwich Y., Gurrea-Soteras M., Sumi N., Joo W.D., Holmberg J.C., Craveiro V., Alvero A.B., Mor G. (2014). Ovulation and extra-ovarian origin of ovarian cancer. Sci. Rep..

[55] Akison L.K., Alvino E.R., Dunning K.R., Robker R.L., Russell D.L. (2012). Transient invasive migration in mouse cumulus oocyte complexes induced at ovulation by luteinizing hormone. Biol. Reprod..

[56] Honda T., Fujiwara H., Yoshioka S., Yamada S., Nakayama T., Egawa M., Nishioka Y., Takahashi A., Fujii S. (2004). Laminin and fibronectin concentrations of the follicular fluid correlate with granulosa cell luteinization and oocyte quality. Reprod. Med. Biol..

[57] Casey R.C., Burleson K.M., Skubitz K.M., Pambuccian S.E., Oegema T.R., Ruff L.E., Skubitz A.P. (2001). Beta 1-integrins regulate the formation and adhesion of ovarian carcinoma multicellular spheroids. Am. J. Pathol..

[58] Chen X., Brewer M.A., Zou C., Campagnola P.J. (2009). Adhesion and migration of ovarian cancer cells on crosslinked laminin fibers nanofabricated by multiphoton excited photochemistry. Integr. Biol..

[59] Yousif N.G. (2014). Fibronectin promotes migration and invasion of ovarian cancer cells through up-regulation of FAK-PI3K/Akt pathway. Cell Biol. Int..

[60] Jayson G.C., Kohn E.C., Kitchener H.C., Ledermann J.A. (2014). Ovarian cancer. Lancet.

