# Supplementary material for: Ovulatory Follicular Fluid Facilitates the Full Transformation Process for the Development of High-Grade Serous Carcinoma
Source: Cancers (Basel). 2021 Jan 26;13(3):468. doi: 10.3390/cancers13030468 (PMC7865564; doi:10.3390/cancers13030468)
Supplement: Supplementary file 1 [file cancers-13-00468-s001.pdf]

Supplementary Materials: Ovulatory Follicular Fluid Facilitates the Full Transformation Process for the Development of High-Grade Serous Carcinoma

Che-Fang Hsu, Pao-Chu Chen, Vaishnavi Seenan, Dah-Ching Ding and Tang-Yuan Chu

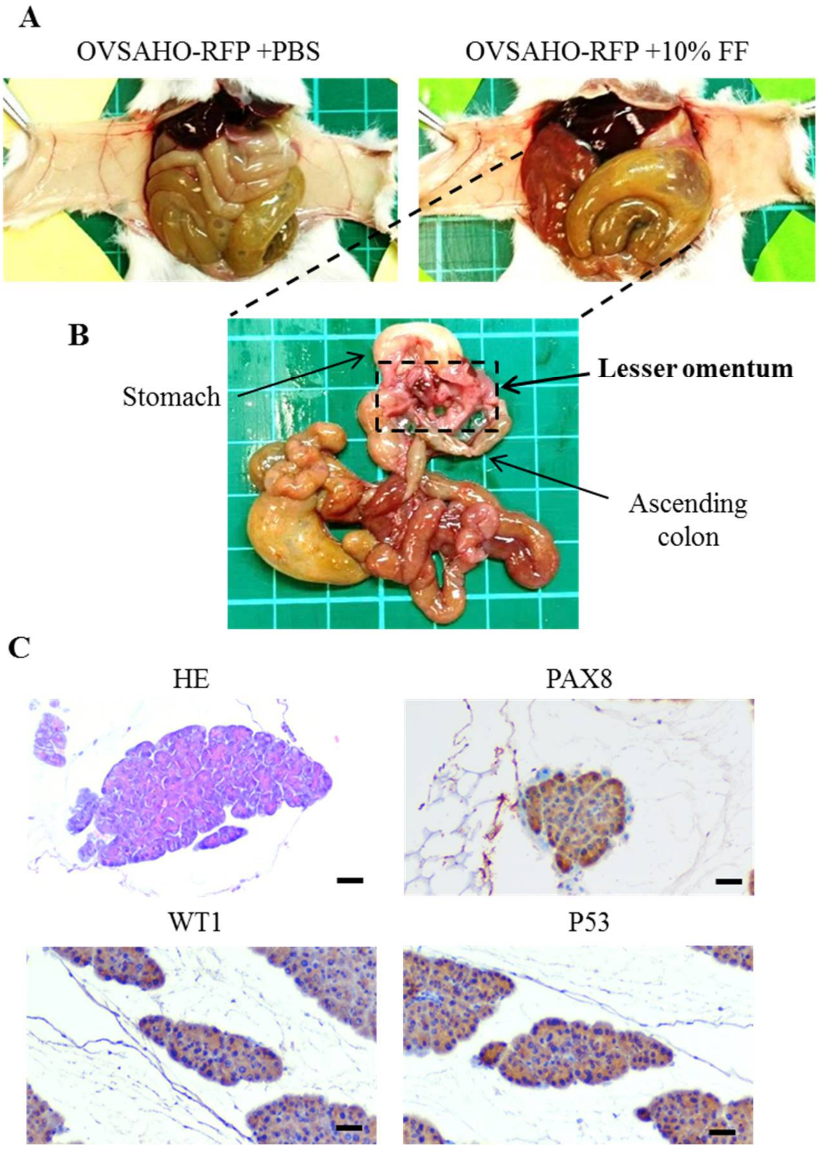

Figure S1. Gross and histochemical characteristics of xenograft tumors of OVSAHO-RFP cells. (**A**) Gross appearance of peritoneal organs showing xenograft tumors of OVSAHO-RFP with or without 10% FF. (**B**) Pictures are shown dissection of the lesser omentum, mesentery, and digestive tract. The background grid is one centimeter. (**C**) Histology of tumor seedings with IHC of PAX8, WT1, and p53. Scale bar = 50 μm.

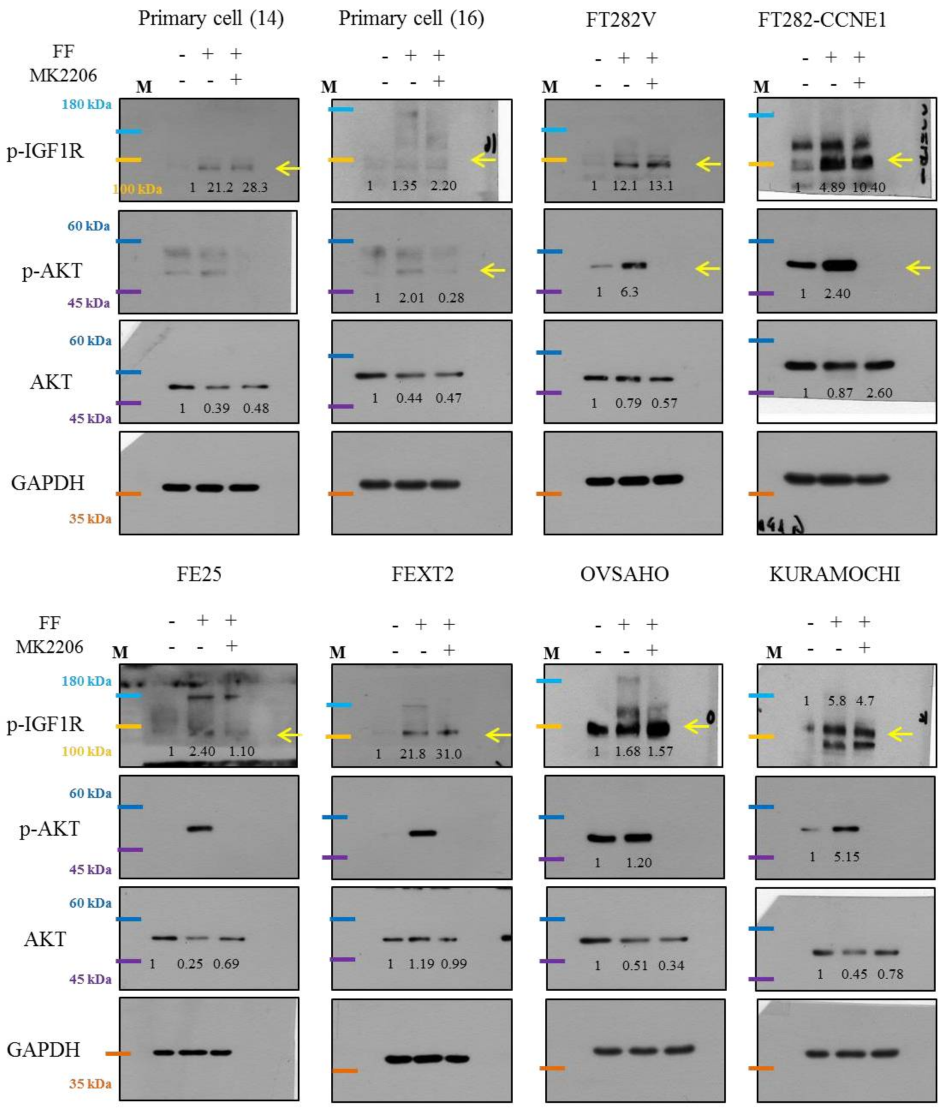

Figure S2. Full Western Blot images for Figure 2A.

Table S1. Continuous stimulation of xenograft incidence and tumor site by follicular fluid.

**Cell Line**	**FE25 +10% FF**	**KURAMOCHI +PBS**	**KURAMOCHI +10% FF**
Harvest	6 months	4.5 months	
Early mortality	4 (25%)	0/5 (0%)	1/5 (20%)
Tumor incidence	4/12 (33%)	5/5 (100%)	4/4 (100%)
Ovary	3/4 (75%)	0/5 (0%)	1/4 (25%)
Peritoneal wall	2/4 (50%)	1/5 (25%)	2/4 (50%)
Mesentery	4/4 (100%)	5/5 (100%)	3/4 (75%)
Liver	1/4 (25%)	0/5 (0%)	3/4 (75%)
Diaphragm	0/4 (0%)	1/5 (25%)	2/4 (50%)
Lesser omentum	0/4 (0%)	1/5 (25%)	4/4 (100%)
Spleen	0/4 (0%)	0/5 (0%)	3/4 (75%)
Ascites (mild)	0/4 (0%)	0/5(0%)	1/4 (25%)

Note: FE25+10% FF (No growth in FE25+PBS)

Table S2. Primary antibodies for WB and IHC.

**Antibody**		**Source (Catalogue No.)**	**Usage & Dilution**
Primary	AKT1	Santa Cruz (sc-5298)	WB 1:200
	p-AKT1	Santa Cruz (sc-52940)	WB 1:200
	p-IGF1R	Genetex (GTX133448)	WB 1:1000
	P53	Cell Signaling (#2527)	IHC 1:100
	Pan-CK	Santa Cruz (sc-15367)	IHC 1:100
	PAX8	Genetex (GTX101583)	IHC 1:100
	WT1	Genetex (GTX15249)	IHC 1:100
	Ki67	BD Biosciences (556003)	IHC 1:100
Secondary	goat anti-rabbit IgG-HRP	Santa Cruz (sc-2030)	WB 1:5000
	goat anti-mouse IgG-HRP	Santa Cruz (sc-2005)	WB 1:5000

Real-Time PCR Primer for EMT Assay

**Genes**		**Sequences**	**NCBI Reference Sequence:**
E-cadherin	Forward	CGG GAA TGC AGT TGA GGA TC	NM_001317185.2
	Reverse	AGG ATG GTG TAA GCG ATG GC	
N-cadherin	Forward	GGC AGA AGA GAG ACT GGG TC	NM_001308176.2
	Reverse	GAG GCT GGT CAG CTC CTG GC	
β-actin	Forward	CAC CAT TGG CAA TGA GCG GTT C	NM_001101.5
	Reverse	AGG TCT TTG CGG ATG TCC ACG T	

Publisher's Note: MDPI stays neutral with regard to jurisdictional claims in published maps and institutional affiliations.

© 2021 by the authors. Licensee MDPI, Basel, Switzerland. This article is an open access article distributed under the terms and conditions of the Creative Commons Attribution (CC BY) license (http://creativecommons.org/licenses/by/4.0/).
